# Dissociating neuronal gamma-band activity from cranial and ocular muscle activity in EEG

**DOI:** 10.3389/fnhum.2013.00338

**Published:** 2013-07-10

**Authors:** Joerg F. Hipp, Markus Siegel

**Affiliations:** ^1^Centre for Integrative Neuroscience, University of TübingenTübingen, Germany; ^2^MEG-Center, University of TübingenTübingen, Germany

**Keywords:** beamforming, electroencephalography, gamma band activity, oscillation, saccadic spike artifact, source analysis, vision

## Abstract

EEG is the most common technique for studying neuronal dynamics of the human brain. However, electromyogenic artifacts from cranial muscles and ocular muscles executing involuntary microsaccades compromise estimates of neuronal activity in the gamma band (>30 Hz). Yet, the relative contributions and practical consequences of these artifacts remain unclear. Here, we systematically dissected the effects of these different artifacts on studying visual gamma-band activity with EEG on the sensor and source level, and show strategies to cope with these confounds. We found that cranial muscle activity prevented a direct investigation of neuronal gamma-band activity at the sensor level. Furthermore, we found prolonged microsaccade-related artifacts beyond the well-known transient EEG confounds. We then show that if electromyogenic artifacts are carefully accounted for, the EEG nonetheless allows for studying visual gamma-band activity even at the sensor level. Furthermore, we found that source analysis based on spatial filtering does not only map the EEG signals to the cortical space of interest, but also efficiently accounts for cranial and ocular muscle artifacts. Together, our results clarify the relative contributions and characteristics of myogenic artifacts confounding visual gamma-band activity in EEG, and provide practical guidelines for future experiments.

## Introduction

With its high temporal resolution, EEG is the most widely used technique for studying the rich temporal dynamics of human brain activity. These dynamics entail neuronal oscillations at various different frequencies (Wang, 2010; Donner and Siegel, 2011; Siegel et al., 2012). Of particular interest are oscillations in the gamma-frequency range (>30 Hz) that reflect local excitatory-inhibitory interactions and are modulated by cognitive processes (Hasenstaub et al., 2005; Bartos et al., 2007; Cardin et al., 2009; Fries, 2009; Donner and Siegel, 2011; Siegel et al., 2012). Although cortical gamma-band activity has been reported with EEG (Tallon-Baudry et al., 1996; Gruber et al., 1999; Müller et al., 2000; Hassler et al., 2011; Hipp et al., 2011; Scheeringa et al., 2011; Plöchl et al., 2012; Muthukumaraswamy and Singh, 2013), the analysis of such activity with EEG is strongly challenged by two types of electromyogenic artifacts (Nunez and Srinivasan, 2010; Muthukumaraswamy, 2013).

First, cranial muscles (facial and neck muscles) induce strong EEG artifacts at frequencies above 30 Hz, which reduce the sensitivity to detect neuronal gamma-band activity (O'Donnell et al., 1974; Goncharova et al., 2003; Whitham et al., 2008). Furthermore, just like neuronal activity, cranial muscle activity can be modulated by cognitive and affective processes (Dimberg et al., 2000; Bradley et al., 2001; Whitham et al., 2008). Thus, varying cranial muscle activity may be mistaken as task-related changes in neuronal gamma-band activity.

Second, ocular muscle activity related to microsaccades impairs the EEG signal in the gamma-frequency range (Yuval-Greenberg et al., 2008). Humans execute about one to two spontaneous microsaccades per second during fixation (Gowen et al., 2007; Martinez-Conde et al., 2009). Ocular muscle contractions at microsaccade onset induce “spike potentials” in the EEG with maximum power in the gamma band at parietal electrodes (Thickbroom and Mastaglia, 1985; Riemslag et al., 1988; Yuval-Greenberg et al., 2008; Keren et al., 2010; Carl et al., 2012). Furthermore, microsaccade rate shows a characteristic suppression-enhancement sequence following visual stimulus transients and is also modulated by cognitive factors (Engbert and Kliegl, 2003; Rolfs et al., 2008; Yuval-Greenberg and Deouell, 2011). Together, spike potentials mimic transient gamma-band activity of neuronal origin, and are thus highly problematic for studying visually gamma-band activity with EEG. However, so far, the spike-potential has only been acknowledged as a short-lived problem following stimulus transients. The effect of this artifact during complex continuous stimuli remains unclear.

In summary, two types of electromyogenic artifacts can confound the EEG. Here, we systematically investigated the effect of these confounds on studying neuronal gamma-band activity with EEG and efficient strategies to cope with these confounds. We found that both, cranial muscle activity and microsaccadic artifacts severely compromise the sensor-level EEG. Using a complex visual motion stimulus, we found that microsaccadic artifacts are not only a short-lived problem following stimulus transients, but may lead to long-lasting signal distortions. We describe efficient strategies to account for electromyogenic artifacts and for successfully studying neuronal gamma-band activity with EEG at the sensor and source level.

## Materials and methods

### Participants, stimuli, and task

**Figure 1:**
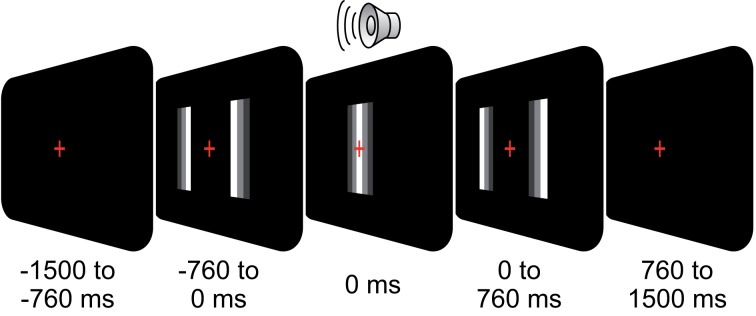
**Behavioral task**. On each trial, subjects fixated a central cross while two moving bars approached each other, overlapped, and diverged again (total duration, 1.52 s). At the moment of overlap (*t* = 0 s), a click-sound was played (duration, 0.02 s). The stimulus was either perceived as two bars passing each other (pass) or bouncing off each other (bounce). Subjects reported their percept via button-press (left/right thumb) after fixation cross offset (0.76 s after stimulus offset).

We recorded EEG in 24 subjects (12 female; mean age: 25; all right handed). All participants had normal hearing, normal or corrected-to-normal vision, and had no history of neurological or psychiatric illness. The study was conducted in accordance with the Declaration of Helsinki and informed consent was obtained from all participants prior to recordings. Subjects were presented with an audio-visual stimulus (500 trials) as described in Figure 1: subjects fixated a central cross while two moving bars approached each other, overlapped, and diverged again (total duration, 1.52 s, size of bars 5 × 0.125° visual angle, starting position at 3.8° eccentricity, velocity: 5°/s). A click-sound (duration: 20 ms, volume: 60 dB SPL) was played at the moment of bar overlap via a central loudspeaker. The stimulus was either perceived as two bars passing each other (pass) or bouncing off each other (bounce). Subjects reported their percept of the ambiguous stimulation via button-press (left and right thumb) after fixation-cross offset. The percept-response mapping was counterbalanced across subjects.

### Data acquisition and preprocessing

This paper presents a re-analysis of data we reported previously (Hipp et al., 2011). We recorded the continuous EEG from 126 scalp sites and the electrooculogram (EOG) from two sites below the eyes all referenced against the nose tip (sampling rate: 1000 Hz; high-pass: 0.01 Hz; low-pass: 250 Hz; Amplifier: BrainAmp, BrainProducts, Munich, Germany; Electrode cap: Electrodes: sintered Ag/AgCl ring electrodes mounted on an elastic cap, Falk Minow Services, Herrsching, Germany). Electrode impedances were kept below 20 kΩ. Offline, the data were high-pass filtered (4 Hz, Butterworth filter of order 4) and cut into trials of 2.5 s duration centered on the presentation of the sound (−1.25 to 1.25 s). First, trials with eye movements, eye blinks, or strong muscle activity were identified by visual inspection and rejected from further analysis (trials retained for further analyses *n* = 345 ± 50, mean ± s.d.). Next, we used independent component analysis (FastICA, http://www.cis.hut.fi/projects/ica/fastica/; Hyvärinen, 1999) to remove artifactual signal components (Jung et al., 2000; Keren et al., 2010). The removed artifactual components constituted facial muscle components (*n* = 45.8 ± 7.84, mean ± s.d.), microsaccadic artifact components (*n* = 1.2 ± 0.82, mean ± s.d.), auricular artifact components (O'Beirne and Patuzzi, 1999) (*n* = 0.5 ± 0.83, mean ± s.d.), and heart beat components (*n* = 0.5 ± 0.59, mean ± s.d.). Alternatively to ICA, we accounted for microsaccadic artifacts by removing confounded data sections identified in the radial EOG using the approach and template described in Keren et al. (2010) (Threshold: 3.5). Importantly, for this analysis step, we did not reject entire trials containing a microsaccadic artifact (79 ± 18%, mean ± s.d., of trials contained at least one saccadic spike artifact), but only invalidated the data in the direct vicinity of detected artifacts (±0.15 s). Whenever the window for time-frequency transform overlapped with invalidated data (see spectral analysis below), it was rejected from further analysis. As a consequence, spectral estimates were based on varying amount of data across time and frequency. We derived the radial EOG as the difference between the average of the two EOG channels and a parietal EEG electrode at the Pz position of the 10–20-system. Notably, rejection based on the radial EOG may miss saccadic spike artifacts of small amplitude that can be detected with high-speed eyetracking (Keren et al., 2010). However, the fact that we did not find any significant saccadic spike artifacts after radial EOG based rejection at those source locations that before cleaning best captured these artifacts (cf. Figure 7C) suggests that potentially remaining artifacts are small.

### Spectral analysis

For time-frequency analyses, we computed spectral estimates using the multi-taper method based on discrete prolate spheroidal (slepian) sequences (Thomson, 1982; Mitra and Pesaran, 1999). We computed spectral estimates across 21 logarithmically scaled frequencies from 5.7 to 181 Hz (0.25 octave steps) and across 19 points in time from −0.9 to 0.9 s (0.1 s steps). We adjusted the temporal and spectral smoothing to match approx. Two hundred and fifty millisecond temporal smoothing and 3/4 octaves spectral smoothing. For frequencies ≥ 16 Hz we used temporal windows of 250 ms and half the number of available tapers for spectral estimates (rounding half the number of tapers to the next lower integer but at least one taper). For frequencies <16 Hz, we adjusted the time window to yield a frequency smoothing of 3/4 octaves with a single taper. We characterized the power response relative to the pre-stimulus baseline at *t* = −0.9 s. To compute estimates of spectral power in the gamma-frequency range for detected saccadic spike events (see above), we employed a Hanning window, a center frequency of 60 Hz, and a bandwidth of 1.5 octaves.

### Source analysis

We used adaptive linear spatial filtering (“beamforming”; Van Veen et al., 1997; Gross et al., 2001) to estimate the spectral power of neural population signals at the cortical source level. In short, for each time, frequency, and source location, 3 orthogonal filters (one for each spatial dimension) were computed that pass activity from the location of interest with unit gain, while maximally suppressing activity from all other sources. The filters were computed separately for each point in time and frequency based on the real part of the cross-spectral density matrix of the data after subtraction of the event-related potential from each single trial. We linearly combined the 3 filters to a single filter in the direction of maximum variance.

**Figure 2:**
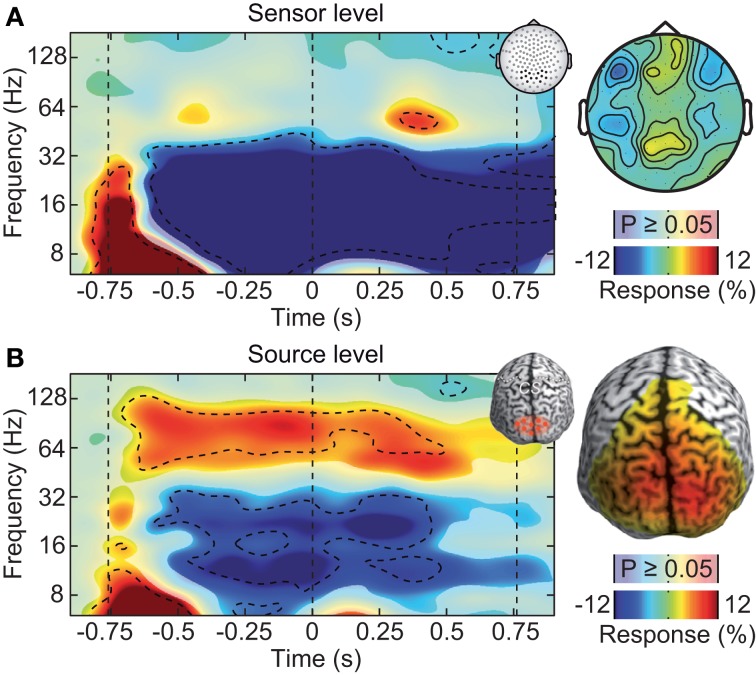
**Neuronal response to the visual-motion stimulus at the sensor and source levels. (A)** Left: power response relative to prestimulus baseline resolved in time and frequency for occipito-parietal electrodes of interest (see inset for electrode positions). Statistically significant differences are indicated by saturated colors (*t*-test, *p* < 0.05), and by contour lines (*t*-test, *p* < 0.05, Bonferroni corrected for time and frequency). Right: scalp topography of power responses in the time-frequency range indicated by the dashed box in the left panel. **(B)** Left: power response relative to prestimulus baseline resolved in time and frequency for an occipito-parietal source region of interest (see inset for region of interest; CS: central sulcus). Right: spatial power distribution in the time-frequency range −0.65 to 0.05 s and 50–110 Hz.

We reconstructed neuronal activity from different sources defined in MNI space: (1) We used 400 locations that homogeneously covered the space below the electrodes with a spacing of 1 cm approximately 1 cm beneath the skull (Hipp et al., 2011). (2) For the source-level analyses of neuronal activity in visual cortex, we used a subset of 8 sources from the set in (1) [center MNI coordinate: (0, −87, 26), see inlet of Figure 2B for locations]. (3) To analyze sources of the saccadic spike artifact, which mimic deep frontal neuronal sources, we used locations on a regular 3D grid of 1 cm spacing that covered the entire brain volume (2014 sources; Figures 5, 7). (4) For the spectro-temporal analysis of the saccadic spike artifact (Figure 7), we used those 20 locations of the 3D grid in (3) with strongest activation during the saccadic spike as assessed in Figure 5B [center MNI coordinate: (−24, 26, −9)].

To derive the leadfield (physical forward model), we first constructed a boundary element head-model from the segmented SPM99/2 template brain. We then averaged the electrode positions measured in 7 subjects and mapped these average positions to MNI space. Finally, we transformed the head-model and electrode positions into the subjects' individual head-space based on individual T1-weigthted structural magnetic resonance images (MRI) and derived the leadfield in the subjects' space. We used the generic MNI-based leadfield for 4 out of 24 subjects for which no MRI was available. The head-model construction was performed using the Matlab toolboxes Fieldtrip (http://www.ru.nl/fcdonders/fieldtrip/; Oostenveld et al., 2011) and SPM (http://www.fil.ion.ucl.ac.uk/spm/).

### Correlation analyses

We quantified the relation between EEG signal power and the saccadic spike rate that we derived from the radial EOG (Keren et al., 2010, threshold: 3.5 s.d.) by correlating these signals within the time range from −0.5 to 0.5 s for each subject. We assessed statistical significance of correlations by Fisher *z*-transforming the correlation values and testing for non-zero correlation across subjects using Student's *t*-test.

### Statistical analyses

All statistical analyses were performed across subjects (random effects analyses). Our analysis capitalized on both, detecting the presence and the absence of effects (e.g., no gamma response on the sensor level in the raw signal, but presence of a gamma response after careful artifact cleaning). To account for these opposing questions, we employed two statistical thresholds: a conservative threshold to identify the presence (*p* < 0.05, Bonferroni corrected for 53.2 effective degrees of freedom corresponding to 19 time points 2.5 fold oversampled times 21 frequency points 3 times oversampled), and a liberal threshold to identify the absence of effects (*p* < 0.05, uncorrected). In Figures 2, 4, 7, and 8 we show these two statistical thresholds in addition to power changes.

### Illustration of sources

We overlaid interpolated beamforming results on the anatomical data from the SPM99/2 template brain at the 2D surface that is spanned by the 400 investigated sources of set (1).

### Analysis software

All data analyses were performed in Matlab (MathWorks, Natick, MA) with custom scripts and open source toolboxes as indicated above.

## Results

We recorded the EEG of 24 healthy subjects performing a perceptual decision task on an ambiguous visual stimulus consisting of two moving bars (Figure 1). The visual stimulation lasted 1.52 s while subjects kept central fixation. Here, we focus on stimulus-driven neuronal activity. For perception related, effects see Hipp et al. (2011).

Large, high-contrast visual motion stimuli, as the one employed here, are known to drive persistent gamma-band activity in the visual cortex (Gray and Singer, 1989; Siegel and König, 2003; Hall et al., 2005; Hoogenboom et al., 2006; Siegel et al., 2007; Hipp et al., 2011; Muthukumaraswamy and Singh, 2013). In contrast, after standard pre-processing (rejection of artifactual trials), the sensor-level analysis of the EEG did not reveal a persistent visual gamma-band response. We analyzed EEG signal power resolved in time and frequency relative to the prestimulus baseline at electrodes above the visual cortex (Figure 2A). Stimulation induced tonic decreases in the theta (5–8 Hz), alpha (8–16 Hz), and beta band (16–32 Hz) (*t*-test, *p* < 0.05, corrected). In the gamma-frequency range, we found only a late, transient increase about 1.1 s after stimulus onset (64 Hz, *t*-test, *p* < 0.05, corrected).

The weak response in the gamma-frequency range at the sensor level stood in strong contrast to the results at the source level (Figure 2B). We employed adaptive linear spatial filtering (beamforming, Van Veen et al., 1997; Gross et al., 2001) to analyze neuronal responses at the cortical source level. We selected a volume of interest in visual cortex. Low-frequency responses were qualitatively similar at the source level as compared to the sensor level. But in contrast to the sensor level, at the source level, stimulation induced a strong and tonic increase of neural activity in the gamma frequency range (64–128 Hz, *t*-test, *p* < 0.05, corrected). Thus, at the source level, the spectro-temporal response closely resembled the expected pattern. We hypothesized that the discrepancy between gamma-band responses at the sensor and source levels was due to electromyogenic artifacts that masked neuronal signals at the sensor level.

**Figure 3:**
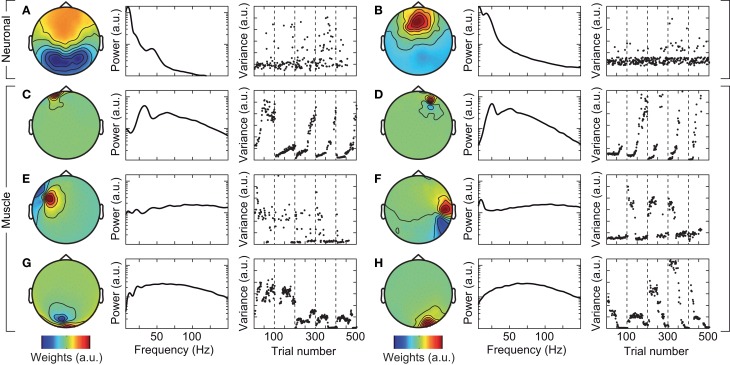
**Exemplary independent components (ICs) that capture neuronal (A,B), and cranial muscle activity (C–H)**. Left: scalp topography of the IC. Center: power spectrum of the IC. Right: signal variance of the IC across the course of the experiment. Examples in left and right columns are taken from different subjects.

**Figure 4:**
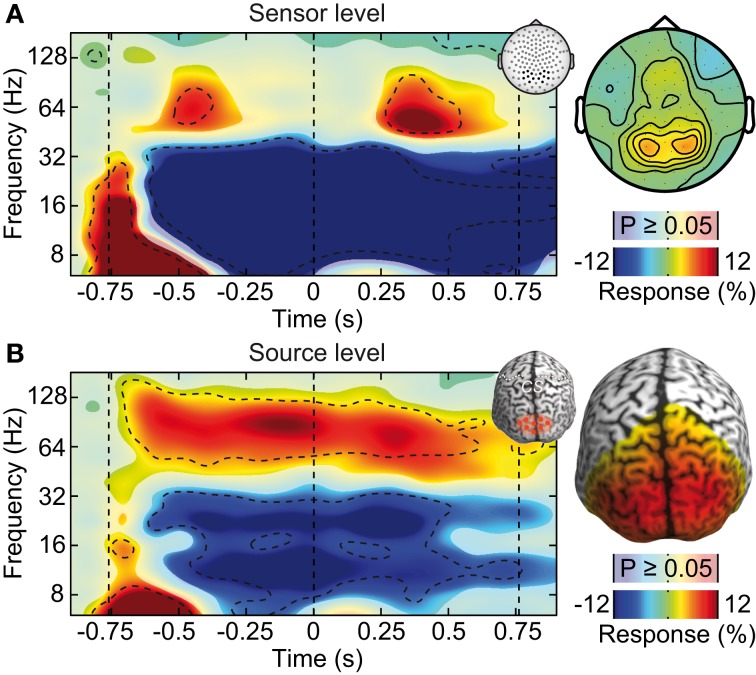
**Effect of ICA-cleaning of cranial muscle artifacts**. Same sensor-level and source-level analysis of stimulus induced responses as displayed in Figure 2. **(A)** Left: power response relative to prestimulus baseline resolved in time and frequency for occipito-parietal electrodes of interest (see inset for electrode positions). Statistically significant differences are indicated by saturated colors (*t*-test, *p* < 0.05), and by contour lines (*t*-test, *p* < 0.05, Bonferroni corrected for time and frequency). Right: scalp topography of power responses in the time-frequency range indicated by the dashed box in the left panel. **(B)** Left: power response relative to prestimulus baseline resolved in time and frequency for an occipito-parietal source region of interest (see inset for region of interest; CS: central sulcus). Right: spatial power distribution in the time-frequency range −0.65 to 0.05 s and 50–110 Hz.

We tested if confounds due to cranial muscle activity were responsible for the missing gamma-band response at the sensor level. We employed independent component analysis (ICA) to decompose the EEG signals into maximally independent components. A substantial fraction of these components captured cranial muscle activity as characterized by highly localized topographies, prominent broad-band signal power above 30 Hz, and strong non-stationarity of signal power across the course of the experiment (see Figure 3 for several exemplary neuronal and artifactual cranial muscle components). Based on these criteria, we identified 46 ± 7.8 (mean ± std) cranial muscle components per subject and removed them from the data. This cleaning procedure reduced the signal power in the gamma frequency range (50–100 Hz; in the baseline interval at −0.9 s) at electrodes above the visual cortex by 62.3%. We then repeated the analysis of stimulus driven changes in signal power for the cleaned data (Figure 4). ICA-cleaning of cranial muscle activity had little effect on the gamma band response at the source-level. By contrast, at the sensor level, we now found two transient power increases in the gamma band around 0.3 and 1.1 s post stimulus onset. Thus, removing cranial muscle activity increased the sensitivity and allowed us for detecting significant changes in gamma-band activity even at the sensor level. However, a salient discrepancy between sensor and source level remained. While at the source level gamma band activity was tonically elevated across the entire stimulation duration, at the sensor level, gamma band activity exhibited two distinct peaks with an intermitted break of more than 500 ms. We next tested if this remaining discrepancy was related to microsaccadic artifacts that have recently been shown to confound gamma-band activity at electrodes above the visual cortex (Yuval-Greenberg et al., 2008).

**Figure 5:**
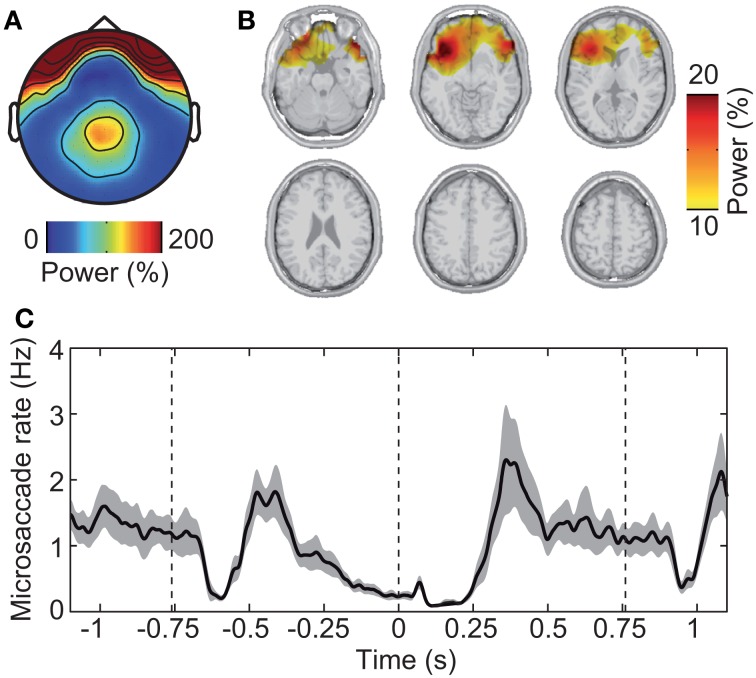
**Saccadic spike artifact that confounds the EEG signal. (A)** Spatial distribution of EEG power in epochs with detected microsaccades (±20 ms) relative to randomly selected epochs without microsaccades. **(B)** Spatial localization of the signal shown in **(A)** using the beamforming source-analysis technique. **(C)** Modulation of microsaccadic spike rate during the experimental trial. The spike rate was estimated using the algorithm of Keren et al. (2010) that is based on filtering and thresholding the radial EOG signal. Gray shading indicates standard error.

First, we investigated the electrical signature of microsaccadic artifacts at the sensor and the source level. We determined microsaccade events based on the radial EOG using the algorithm introduced by Keren et al. (2010). The mean microsaccade rate was 1.18 ± 0.289 Hz (mean ± s.d. across subjects). The topographical distribution of EEG gamma power around the detected microsaccades was characterized by a global maximum at frontal electrodes and a local maximum at parietal electrodes (Figure 5A). This corresponds well with previous investigations of the spike potential (Yuval-Greenberg et al., 2008; Keren et al., 2010; Carl et al., 2012). Microsaccadic EEG artifacts originate from the contraction of ocular muscles at microsaccade onset (Thickbroom and Mastaglia, 1985; Yuval-Greenberg et al., 2008; Carl et al., 2012). Indeed, beamforming constrained to the brain volume localized gamma power around detected microsaccades to orbito-frontal regions in the vicinity of ocular muscles (Figure 5B).

Second, we investigated the time-course of microsaccade occurrence. We found that the rate of microsaccades was strongly modulated throughout the experimental trial (Figure 5C). Immediately after stimulus onset (−0.76 s), microsaccade rate dropped from ~1 Hz baseline level to near 0 (~–0.6 s) followed by a rebound (~–0.4 s). After this initial transient response, the rate steadily dropped for an extended period of time (~–0.3 to 0.3 s), rebounded (~0.4 s), and stayed near baseline-level until after stimulus offset. There, the rate dropped again transiently followed by a rebound and overshoot (~1.0 s).

In summary, the spike potential strongly affected gamma-power at EEG sensors overlying parietal visual cortex. But in source space, the saccadic spike artifact spatially well-separated from the visual cortex. Moreover, visual stimulation was accompanied by a complex temporal modulation of microsaccade rate. Thus, microsaccadic artifacts may have confounded the sensor level estimate—but not the source level estimate—of neuronal activity in visual cortex in a complex fashion. To test this, we next investigated the effect of removing microsaccade artifacts from the EEG.

**Figure 6:**
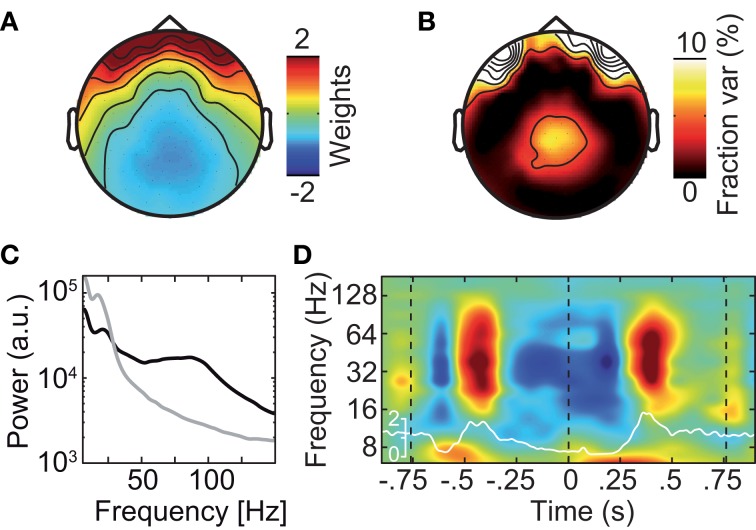
**Exemplary independent component (IC) that captures the saccadic spike artifact. (A)** Scalp topography of the saccadic spike IC. **(B)** Scalp topography of the fraction of signal power explained by the saccadic spike IC. **(C)** Power spectrum of the saccadic spike IC (black) and average power spectrum of all physiological (i.e., non-muscle) ICs (gray). **(D)** Change in the saccadic spike ICs' signal power relative to prestimulus baseline resolved in time and frequency. The white line shows the concurrent microsaccade rate for comparison (compare Figure 5A).

**Figure 7:**
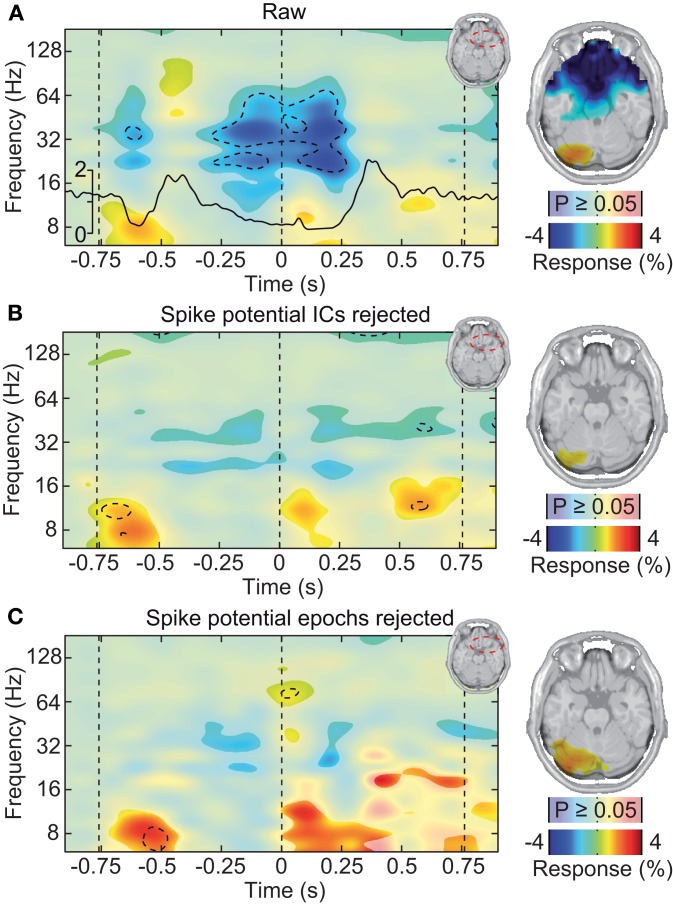
**Saccadic spike artifact at the source level. (A)** Left: change in signal power relative to prestimulus baseline in a frontal volume of interest in the vicinity of the ocular muscles (see upper right inset). The black line shows the concurrent microsaccade rate (compare Figure 5A). Statistically significant differences are indicated by saturated colors (*t*-test, *p* < 0.05), and by contour lines (*t*-test, *p* < 0.05, Bonferroni corrected for time and frequency). Right: spatial distribution of signal power in the time-frequency range −0.25 to 0.25 s and 30–70 Hz, overlaid on an axial slice. EEG signals were cleaned for muscle activity but not for microsaccadic artifacts. **(B)** Same as in **(A)**, but after removal of microsaccadic artifacts using ICA. **(C)** Same as in **(A)**, but after removal of microsaccadic artifacts using epoch rejection.

Different approaches have been described to account for microsaccadic artifacts in EEG. We used two complementary approaches. First, we rejected data epochs with identified microsaccades from further analysis (Keren et al., 2010). Second, we employed ICA to remove spike potential components form the data (see Figure 6 for an exemplary saccadic spike artifact component; 1.2 ± 0.82 components removed per subject) (Keren et al., 2010; Hassler et al., 2011; Plöchl et al., 2012). The removed ICA components accounted for about 4.5% of the total signal power in the gamma frequency range (50–100 Hz; in the baseline interval at −0.9 s) at electrodes above the visual cortex (~10.7% of signal power for the data cleaned for muscle artifacts). To test both cleaning procedures, we instigated their effect on the best estimate of the isolated artifactual signal, i.e., the above described modulation of signal power close to the eyeballs (see Figure 5B). Before accounting for microsaccadic artifacts, signal power in the gamma band was strongly modulated by saccade rate (Figure 7A; Correlation analysis, 50–100 Hz, −0.5 to 0.5 s, *r*^2^ = 0.279, *t*-test, *p* = 9.9 × 10^−8^). Using either one of the cleaning procedures, the modulation by saccade rate in the source volume was negligible (Figures 7B,C; Correlation analyses, 50–100 Hz, −0.5 to 0.5 s, ICA cleaned: *r*^2^ = 0.041, *t*-test, *p* = 0.018; microsaccade epoch rejection: *r*^2^ = 2.70 × 10^−5^, *t*-test, *p* = 0.955). Thus, both cleaning procedures well-accounted for the saccadic spike artifact.

**Figure 8:**
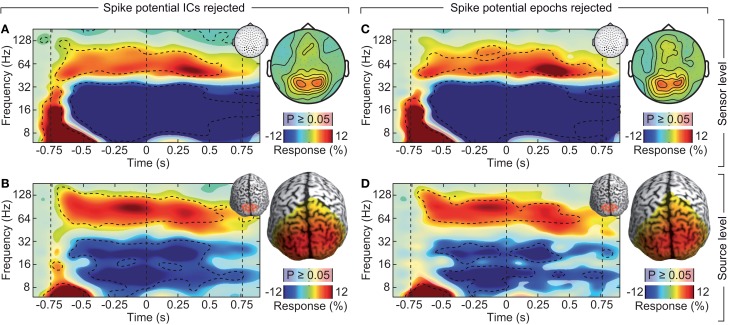
**Effect of rejecting cranial muscle artifacts and saccadic spike artifacts**. Same sensor-level and source-level analysis of stimulus induced responses as displayed in Figures 2, 4. **(A,C)** Left: power response relative to prestimulus baseline resolved in time and frequency for occipito-parietal electrodes of interest (see insets for electrode positions). Statistically significant differences are indicated by saturated colors (*t*-test, *p* < 0.05), and by contour lines (*t*-test, *p* < 0.05, Bonferroni corrected for time and frequency). Right: scalp topography of power responses in the time-frequency range −0.65 to 0.05 s and 50–110 Hz. **(B,D)** Left: power response relative to prestimulus baseline resolved in time and frequency for an occipito-parietal source region of interest (see insets for the region of interest; CS: central sulcus). Right: spatial power distribution in the time-frequency range indicated by the dashed box in the left panels.

If the saccadic spike artifact caused the remaining discrepancy between sensor and source level activity in response to visual stimulation (see Figure 4), applying the cleaning procedures should resolve this discrepancy. Indeed, while the raw sensor level gamma band power above the visual cortex was strongly modulated by the saccade rate (see Figure 4A; Correlation analysis, 50–100 Hz, −0.5 to 0.5 s, *r*^2^ = 0.283, *t*-test, *p* = 9.9 10^−7^), using either one of the cleaning procedures revealed un-modulated persistent activity (Figures 8A,C; Correlation analyses, 50–100 Hz, −0.5 to 0.5 s, ICA cleaned: *r*^2^ = 0.0048, *t*-test, *p* = 0.513; microsaccade epoch rejection: *r*^2^ = 0.0087, *t*-test, *p* = 0.190). As a consequence, the estimate of visually driven gamma power at the sensor level was now qualitatively and quantitatively similar to the source level estimates (cf. Figures 2B, 4B, 8B,D; Correlation analyses, 50–100 Hz, −0.5 to 0.5 s, for all: *r*^2^ < 0.0078, *t*-test, *p* > 0.172). Thus, carefully accounting for both cranial and ocular muscle artifacts uncovers visual gamma band activity at the sensor level comparable to the source level.

**Figure 9:**
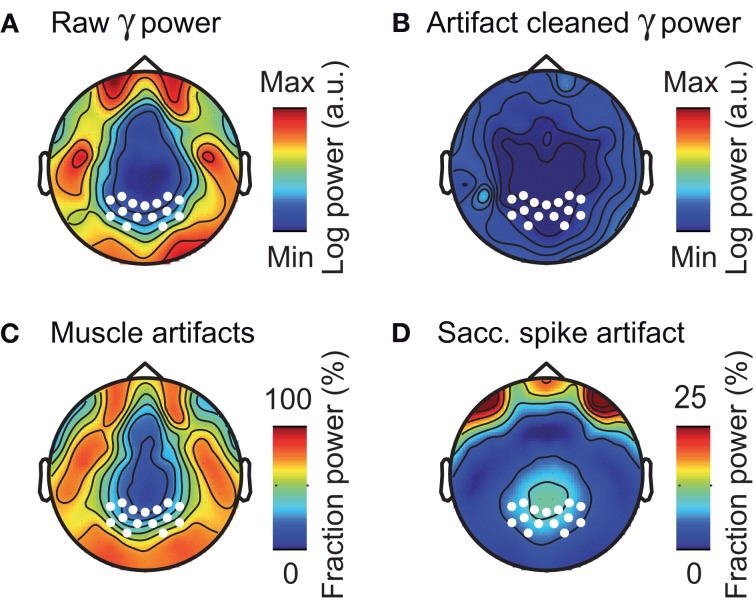
**Composition of EEG activity in the gamma-band. (A)** Scalp distribution of raw pre-stimulus power in the gamma frequency band (50–100 Hz) without ICA cleaning or spike potential rejection. White marks indicate the location of the occipito-parietal electrodes used in all other sensor-level analyses. **(B)** Scalp distribution of pre-stimulus gamma power for artifact cleaned data (ICA based removal of saccadic spike and muscle artifacts). **(C,D)** Scalp topography of the fraction of pre-stimulus gamma power that was removed by ICA cleaning of muscular- and saccadic spike artifacts, respectively.

The above analyses focused on electrodes overlying the visual cortex. However, the problem of muscle artifacts may even be stronger for EEG electrodes directly overlying muscles (Goncharova et al., 2003; Whitham et al., 2008). Thus, we next investigated the topography of muscle artifacts in the gamma frequency range (50–100 Hz) across the entire scalp (Figure 9). The raw EEG gamma power was characterized by a prominent belt-like structure that contained frontal, temporal, and occipital electrodes above the major cranial muscles (Figure 9A). Removing muscle and saccadic spike artifacts using ICA dramatically reduced the gamma power at these electrodes (Figure 9B). The reduction in signal power due to removing muscular artifacts was up to 75% at frontal, temporal, and occipital electrodes (Figure 9C). The reduction in signal power due to removing saccadic spike artifacts was up to 33% at frontal electrodes and about 10% at occipito-parietal electrodes (Figure 9D). In summary, in accordance with previous reports (Goncharova et al., 2003; Whitham et al., 2008), we found muscular artifacts in EEG to have a characteristic topography that is neither restricted to, nor strongest at electrodes above the visual cortex.

**Figure 10:**
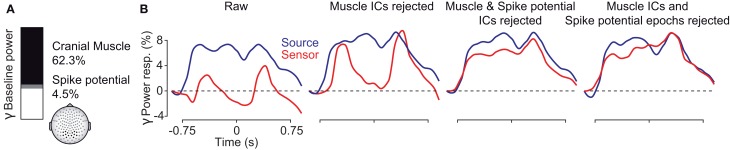
**Summary of findings. (A)** Fraction of pre-stimulus gamma power at EEG sensors above the visual cortex related to extracranial muscle activity (black), saccadic spike potentials (SP, gray), and putative neuronal activity (white) in the baseline interval at −0.9 s. **(B)** Temporal evolution of visually driven gamma-band activity at the sensor (red) and source (blue) level. The four plots show the signals at different stages of artifact cleaning.

To conclude, we systematically investigated the EEG signal in response to a long-lasting, non-stationary visual stimulus that is known to drive persistent gamma-band activity. The results are summarized in Figure 10. At electrodes above the visual cortex, cranial and ocular muscle activity contributed as much as 2/3 of the total signal power in the gamma frequency range (Figure 10A). These artifacts strongly affected the sensitivity to detect visually driven neuronal gamma-band activity and effectively drowned neuronal gamma-band activity in noise (Figure 9B, left, red line). Removisng cranial muscle artifacts using ICA substantially improved sensitivity but transient microsaccade-related artifacts still distorted the estimate of neuronal activity (Figure 10B, center left, red line). Only accounting for both, cranial and ocular muscle artifacts, revealed sustained gamma-band activity at the sensor level (Figure 10B, center right and right, red lines). Alternatively, an analysis in source space using beamforming accounted for a large part of cranial and ocular myogenic artifacts (Figure 10B, left, blue line). Additional artifact cleaning only moderately improved the analysis at the source level (Figure 10B, blue lines). Taken together, our results show that if carefully accounted for both, cranial and ocular muscle artifacts, the EEG allows for studying persistent visual gamma band activity at the sensor level. Alternatively, an analysis in source space not only maps the signals to the space of interest, but also largely accounts for cranial and ocular muscle artifacts.

## Discussion

Our results systematically show the relative contributions and characteristics of different myogenic artifacts on the sensor and source level EEG. In particular, our results underline the necessity to carefully clean EEG data for studying neuronal gamma-band activity. Not accounting for myogenic artifacts may lead to false conclusions, such as e.g., missing an effect (cf. Figure 2) or misinterpreting a continuous activation as transient events (cf. Figure 4). However, if artifacts are carefully accounted for, fast oscillations can be well-studied with EEG.

### Cranial muscle artifacts

Cranial muscles generate electrical activity in the gamma frequency-range that confounds neuronal EEG signals (Goncharova et al., 2003; Whitham et al., 2008). Here, we investigated the effect of cranial muscle activity in the context of a visual motion stimulus that is known to induce persistent neuronal responses in the gamma band. We found that cranial muscle artifacts were so strong that no proper neuronal gamma-band response was detected in the sensor-level EEG. Only after accounting for muscle artifacts by ICA or analysis in source space, we were able to recover a sustained neuronal gamma-band response. The visual stimulus employed here, was not tailored to drive maximal gamma-band responses. It is possible that visual stimuli specifically designed to maximize gamma-band responses, such as e.g., large circular gratings (Muthukumaraswamy and Singh, 2013; Hoogenboom et al., 2006), may lead to responses visible on the sensor level even without muscle cleaning.

The poor sensitivity of the sensor-level EEG for neuronal gamma-band activity in face of cranial muscle activity likely underlies a general skepticism about the possibility to study neuronal gamma-band activity with EEG (Whitham et al., 2008; Nunez and Srinivasan, 2010). This poor sensitivity also may have led to conflicting results of previous studies on visual gamma-band activity using visual motion stimuli. While some studies reported visual gamma-band responses (Gruber et al., 1999; Müller et al., 2000), others found responses only for some stimulus conditions (Müller et al., 1996), or not at all (Juergens et al., 1999). More recent EEG studies that employed ICA cleaning consistently reported gamma-band responses to visual motion stimuli (Hipp et al., 2011; Scheeringa et al., 2011; Plöchl et al., 2012) that resemble gamma-band responses observed invasively in animals (Gray and Singer, 1989; Kreiter and Singer, 1992; Siegel and König, 2003; Nase et al., 2003) and humans (Lachaux et al., 2005), and non-invasively in the human MEG (Hall et al., 2005; Hoogenboom et al., 2006; Siegel et al., 2007, 2008). Our findings corroborate these studies by directly comparing visually driven gamma-band responses in EEG with and without accounting for cranial muscle artifacts. The topography of muscular artifacts in the EEG signal (see Figure 9) suggests that these artifacts are problematic for a wide range of applications. Future studies will need to investigate to what extent the present results transfer to other types of neuronal gamma-band activity, such as e.g., auditory or motor related gamma-band responses.

### Saccadic spike artifact

In addition to cranial muscle activity, ocular muscle activity at the onset of microsaccades—the saccadic spike potential—has been identified as a problematic myogenic artifact that affects parietal gamma-band activity in EEG (Yuval-Greenberg et al., 2008). This artifact has previously been acknowledged as a transient problem in response to stimulus onset (Yuval-Greenberg et al., 2008; Keren et al., 2010; Hassler et al., 2011; Schwartzman and Kranczioch, 2011; Yuval-Greenberg and Deouell, 2011). Our results show that, for non-stationary stimulation and cognitive demands, the saccadic spike artifact is not limited to a transient signal distortion, but can induce a persistent modulation of gamma-band activity. While the transient changes following the onset and the offset of the stimulus are comparable to the known artifact, we also found a suppression persisting for more than 600 ms, which seems to be of qualitatively different nature. Our finding of complex dynamics of spike artifacts is in line with previous observations. Microsaccade rate is not only modulated by stimulus onset (Engbert and Kliegl, 2003; Rolfs et al., 2008), but also by cognitive processes such as attention (Hafed and Clark, 2002; Engbert and Kliegl, 2003; Gowen et al., 2005; Laubrock et al., 2005) or in a visual oddball task (Valsecchi et al., 2007).

In summary, our findings show that for an experimental task with non-stationary stimulation and cognitive demands, the saccadic spike artifact can be more problematic and far-reaching than previously acknowledged. Future experiments need to disentangle the contributions of non-stationary stimulation (bottom–up input), cognitive demands (top–down processing), and their possible interaction to the complex temporal modulation of saccade rates. A first indication if microsaccadic artifacts constitute a problem in an EEG experiment may be derived from the saccade rate that can be extracted from the radial EOG (Keren et al., 2010) or from concurrent eye tracking.

### Removing myogenic artifacts from EEG

We explored two different approaches to remove myogenic artifacts from the sensor-level EEG: ICA and epoch rejection.

ICA has proven to be a useful tool to account for various non-neuronal artifacts (Jung et al., 2000; Shackman et al., 2009; Keren et al., 2010; McMenamin et al., 2010; Hipp et al., 2011; Hassler et al., 2011; Plöchl et al., 2012). Here, we successfully used ICA to account for cranial and ocular muscle artifacts. However, unresolved issues remain with this approach. Although there have been several attempts for automatic or semi-automatic detection of artifactual components (Delorme et al., 2007; Mammone and Morabito, 2008; Mantini et al., 2008; Viola et al., 2009), there is no accepted standard procedure. This leaves a subjective component to ICA-based cleaning procedures. Furthermore, there is no guarantee that ICA fully separates artifactual from non-artifactual components. Incomplete separation may either lead to a reduction of the physiological signal, if artifact components that also include neuronal signals are removed, or may lead to a suboptimal noise level, if neuronal components that also include artifacts are kept. The present study well-illustrates these problems. For 6 out of 24 subjects, we could not identify an independent component that unequivocally represented the microsaccadic spike artifact. In such situations, more advanced variants of ICA may improve artifact separation and removal (see e.g., Hassler et al., 2011). Despite these limitations, the present study corroborates the usefulness of ICA for EEG artifact removal.

Alternatively to ICA, data sections affected by artifactual signals can be rejected from the analysis. For muscle artifacts, this is typically done automatically or manually by identifying epochs with salient high-frequency activity. We also applied this approach to the present data. To remove saccadic spike artifacts, we detected spike potentials using the radial EOG (Keren et al., 2010) and rejected the corresponding EEG epochs from the analysis. While the epoch rejection approach may be more conservative than ICA, it reduces the amount of data for analysis. Similar as ICA, there is also a subjective component either by manually selecting epochs or by adjusting artifact thresholds. For the saccadic spike artifact, we found that the rejection approach alone can sufficiently remove artifacts. For cranial muscle artifacts the situation was very different, because such artifacts to a variable degree confound all data. Accordingly, we found that epoch rejection can only account for the strongest cranial muscle contractions, but did not suffice to analyze fast oscillatory activity at the EEG sensor level. Thus, to account for cranial muscles, a combination of epoch rejection followed by ICA cleaning as demonstrated here is advisable.

### Source analysis

Our results suggest source-analysis based on beamforming as an efficient alternative to account for myogenic artifacts when studying visual gamma-band activity with EEG. We found that beamforming does not only map the signal to the cortical space of interest, but also effectively accounts for both, cranial muscle activity and the saccadic spike artifact. Thus, the spatial beamforming filters are capable of efficiently separating intracranial neuronal sources from extracranial muscle activity. Here, we studied stimulus driven gamma-band activity in the visual cortex. Future studies need to determine to what extent beamforming also efficiently separates other forms of gamma-band activity from extracranial muscle activity.

In general, our results advocate analyzing EEG gamma-band activity at the cortical source level. Given the ill-posed nature of estimating cortical source activity from surface recordings, there is good reason to critically evaluate findings based on source analyses. As a consequence, for many researchers, the sensor level data is the gold standard. However, our results demonstrate that this, at first sight, conservative attitude may lead to false conclusions. Our data provide an example, where the beamforming results at the source-level are clear-cut, while the sensor level results are not. In fact, the sensor-level results may have led to false conclusions such as strongly non-stationary or absent neuronal gamma-band responses. Thus, the valid skepticism toward source analysis should not lead to a general refusal of source analysis and to taking the sensor level data as the ground truth without any skepticism.

We used beamforming for source analysis (Van Veen et al., 1997; Baillet et al., 2001; Gross et al., 2001; Hall et al., 2005), which aims at estimating neuronal activity at a source location of interest, while maximally suppressing activity from other sources. It remains to be investigated, how our findings generalize to other source analysis techniques such as e.g., minimum norm (Hämäläinen and Ilmoniemi, 1994), LORETA (Pascual-Marqui et al., 1994), or equivalent current dipole fitting.

In summary, our results suggest that for investigating gamma-band activity in the visual cortex, beamforming analysis in source space does not only provide spatial specificity, but also efficiently accounts for cranial muscle and microsaccadic spike artifacts.

### Conflict of interest statement

The authors declare that the research was conducted in the absence of any commercial or financial relationships that could be construed as a potential conflict of interest.
